# The effects of image compression on quantitative measurements of digital panoramic radiographs

**DOI:** 10.4317/medoral.17912

**Published:** 2012-08-28

**Authors:** Füsun Yasar, Burcu Apaydın, Hasan-Hüseyin Yılmaz

**Affiliations:** 1 Assistant Professor Doctor, Selcuk University Faculty of Dentistry Oral Radiology Department, Kampüs Konya Turkey; 2 Research Assistant, Selcuk University Faculty of Dentistry Oral Radiology Department, Kampüs Konya Turkey; 3 Professor Doctor, Department of Oral Radiology Sifa University Faculty of Dentistry Izmir Turkey

## Abstract

Objectives: The aims of this study were to explore how image compression affects density, fractal dimension, linear and angular measurements on digital panoramic images and assess inter and intra-observer repeatability of these measurements. Study Design: Sixty-one digital panoramic images in TIFF format (Tagged Image File Format) were compressed to JPEG (Joint Photographic Experts Group) images. Two observers measured gonial angle, antegonial angle, mandibular cortical width, coronal pulp width of maxillary and mandibular first molar, tooth length of maxillary and mandibular first molar on the left side of these images twice. Fractal dimension of the selected regions of interests were calculated and the density of each panoramic radiograph as a whole were also measured on TIFF and JPEG compressed images. Intra-observer and inter-observer consistency was evaluated with Cronbach’s alpha. Paired samples t-test and Kolmogorov-Smirnov test was used to evaluate the difference between the measurements of TIFF and JPEG compressed images. Results: The repeatability of angular measurements had the highest Cronbach’s alpha value (0.997). There was statistically significant difference for both of the observers in mandibular cortical width (MCW) measurements (1st ob. p: 0.002; 2nd ob. p: 0.003), density (p<0.001) and fractal dimension (p<0.001) between TIFF and JPEG images. There was statistically significant difference for the first observer in antegonial angle (1st ob p< 0.001) and maxillary molar coronal pulp width (1st ob. p<0.001) between JPEG and TIFF files. Conclusions: The repeatability of angular measurements is better than linear measurements. Mandibular cortical width, fractal dimension and density are affected from compression. Observer dependent factors might also cause statistically significant differences between the measurements in TIFF and JPEG images.

** Key words:**Digital panoramic radiography, image compression, linear measurements, angular measurements, fractal dimension.

## Introduction

Intraoral and extraoral dental radiography is an integral part of dental task. Dental radiographs are used from diagnosis and treatment plan of various lesions and conditions within the jaws to follow up the success of treatments such as root canal therapy, implant applications, orthognathic surgery or periodontal therapy. There is a transition from conventional radiography to digital radiography in both intra-oral and extra-oral techniques. There are patients seeking for treatment with their digital radiographs, exposed in various clinics and recorded to compact discs in JPEG formats. As a result of this, it is necessary to investigate the effects of compression on various properties of radiographs. Panoramic imaging which is also called pantomography is a single tomographic image of the facial structures that includes both the maxillary and mandibular dental arches and their supporting structures (1). Panoramic films are useful for evaluating skeletal and dental pathology, making dimensional assessments and determining relative angulations of teeth with other structures (2). Digital panoramic radiography is gradually replacing conventional panoramic radiography. Different kinds of measurements, composed of linear and angular measurements are performed on panoramic images (1). With the advent of digital panoramic imaging, making measurements with image analysis programs became easier.

The gonial angle is an important angle of the craniofacial complex and it is significant for the diagnosis of craniofacial disorders (2). The antegonial notch or angle is the depression along the jawline and it is the upward curving of the inferior border of the mandible anterior to the gonion. It lies at the junction of body and the ramus (3). The strategic position of the shape has been considered to be an indicator of how the mandible will grow (4). Mandibular cortical width is used as an indicator of bone loss in various studies (4).

Age calculation became increasingly important in forensic sciences and different techniques for dental age calculation with radiographs are reported. The maximum tooth length and coronal pulp width measurements are used in age estimation in the method of Kvall et al (5). Fractal dimension has also been used to evaluate trabecular bone architecture (6,7).

Because of the bigger file size of digital panoramic radiographs, the effects of size reduction through compression on digital measurements on panoramic radiographs is an important issue that needs to be investigated for possible effects on image quality (8). The reduced image size will contribute not only to electronic space but also to a faster data transmission (9,10).

In this study gonial angle, antegonial angle, mandibular cortical width, coronal pulp widths of maxillary and mandibular first molar teeth, tooth lengths of maxillary and mandibular first molar, mean density of panoramic radiographs and fractal dimension were evaluated in TIFF and JPEG compressed images separately. It was aimed to assess the repeatability of these measurements and explore how image compression affects density, fractal dimension, linear and angular measurements performed on digital panoramic images.

## Material and Methods

**Figure 1:**
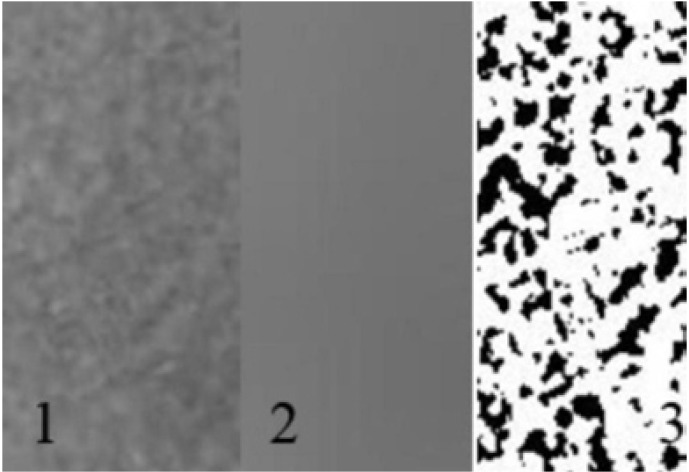
1) The original region of interest which was created for fractal dimension calculations. 2) Blurred form of the region of interest by applying a Gaussian filter with a diameter of 20. 3) Binary form of the region of interest with which fractal dimension was calculated.

One dentomaxillofacial radiologist evaluated the consecutive images to determine its quality for patient positioning, head alignment, film density -contrast- artifacts and images that did not achieve these standards were not included to the study. The radiographs of patients having fractures, pathological entities and systemic diseases affecting bone, skeletal deformities were excluded from the study. All of the radiographs included to the study should have caries/restoration/prosthetic crown free maxillary and mandibular first molar teeth on the left side of the jaws (on the right side of the panoramic images) and the teeth should be clearly visible. Sixty one digital panoramic images were used in the study meeting the above mentioned criteria. None of the radiographs were exposed for this study but they were chosen among previously exposed radiographs for other purposes. This study was approved by the Ethic Committee of Selcuk University Dentistry Faculty with a number of 2011/04-25. All of the measurements (coronal pulp widths of maxillary and mandibular first molar teeth and the total lengths of these teeth; mandibular cortical width; antegonial angle; gonial angle, fractal dimension) were performed on the left side of the jaws on panoramic radiographs and the density of the panoramic images were measured as a whole for each image. ImageJ which is a public domain program was used for all measurements and fractal dimension calculations. This program can be downloaded from http://rsb.info.nih.gov/ij/. Fractal dimension was measured in Box Counting method. The panoramic images included to the study were exposed with the same digital panoramic machine and by the same operator (Kodak 8000 with a charge-coupled device sensor).The radiographs which were chosen for the study were exported as TIFF files from Kodak software. Afterwards, high JPEG compression was applied to these original TIFF images and a second group of images in JPEG format were obtained. Low quality with baseline optimized format (quality number: 3) was used in compression by using Adobe Photoshop CS2 program. Region of interests (ROI) were created in the retromolar region of the mandible on the left side of original TIFF images (Fig. 3 –labeled as 5) and great care was shown not to include periodontal ligament space, lamina dura, root structure and mandibular canal. These ROIs were also compressed in JPEG format in low quality with baseline optimized format (quality number: 3). Fractal dimension was measured both on original TIFF images and JPEG compressed images in a similar way described in other studies (7,8). (Fig. 1- labeled as 1). Each pixel has a mean gray value which is expressed with numbers in digital images, so during image processing and analyzing processes, it is possible to apply mathematical operations to the image at pixel base such as multiply, add, subtract. At first, the region of interest was blurred by a Gaussian filter having a radius of 20 (Fig. 1- labeled as 2), later this blurred version was subtracted from the original one and 128 was added to this newly created image at pixel base. By adding 128 to each pixel, the opacity of the image increases or in other words mean gray value of the image increases. After this, the image was made binary with ImageJ (Fig. 1- labeled as 3) and the fractal dimension in box-counting method of this binary image was calculated.

**Figure 2:**
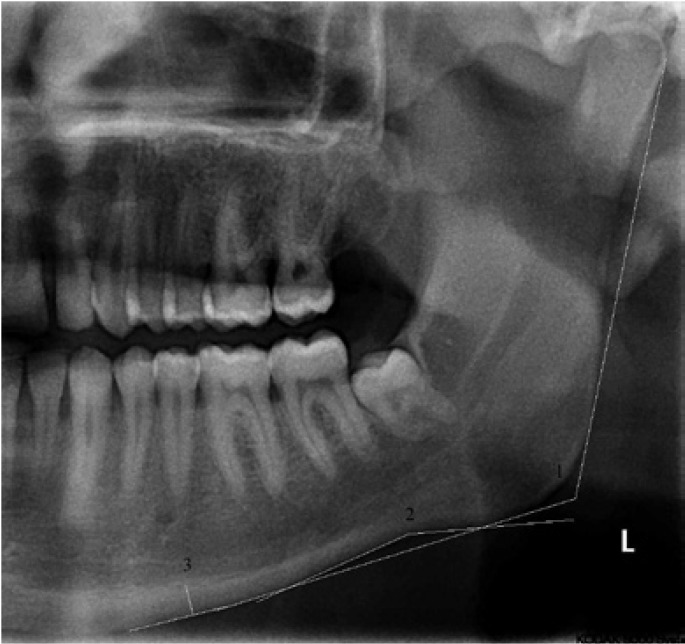
Cropped panoramic radiograph showing the gonial angle (1), antegonial angle (2) and mandibular cortical width (3).

**Figure 3:**
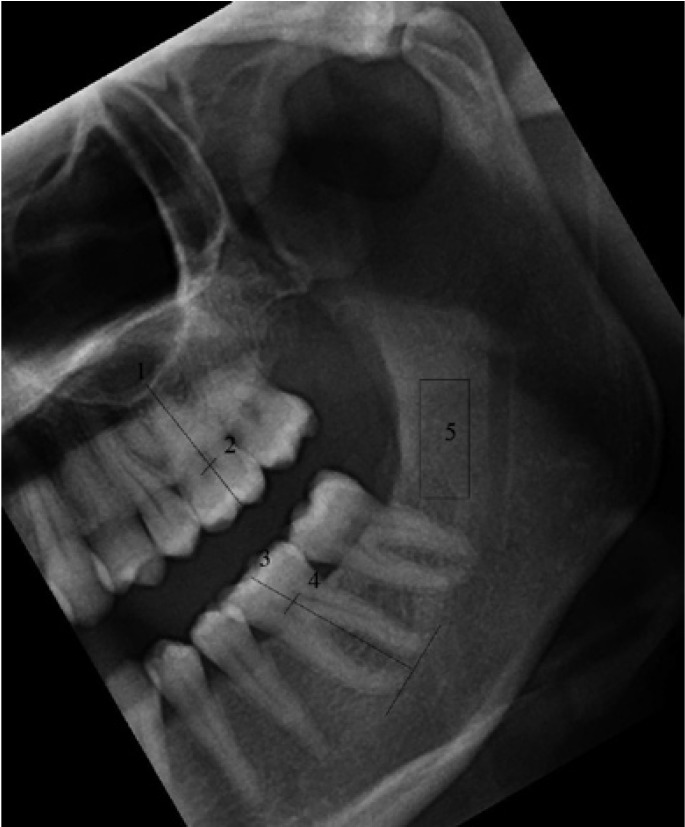
Cropped and rotated digital panoramic radiograph showing the measurement of maxillary (2) and mandibular (4) coronal pulp width, maxillary (1) and mandibular (3) tooth length and the region of interest placement (5) for fractal dimension calculations.

Before the observations began, the two observers measured all of the parameters used in the study, except fractal dimension and density, together on 10 digital panoramic radiographs by overviewing their definition from reference articles especially for gonial and antegonial angle, and mandibular cortical width (2,3,11); and those radiographs were not included to the study. The two observers measured the above mentioned parameters on 61 digital panoramic radiographs in original TIFF images and JPEG compressed images twice separately. There was two weeks interval between the measurements of original TIFF files and JPEG compressed files. All of the measurements were repeated four weeks after the first measurements finished. The observers were not allowed to make any brightness/contrast adjustments and both of them evaluated the radiographs under the same conditions. Digital images were viewed in a silent and dim room with a 19 inch high-resolution (1280 X 1024 pixels and 256 grey levels-super video graphics array) color liquid crystal monitor. Both of the observers were working in the same Maxillofacial Radiology clinic however their working time was different from each other. The first observer was working as an oral radiologist for more than 10 years and the second observer was working in the same clinic for one year. Fractal dimension of the selected region of interests and density of the panoramic radiographs were measured only once by one of the observers in JPEG and TIFF images because, fractal dimension and density will be same when the same ROI or image is used. The following measurements were performed by the two observers separately:

1. Gonial angle (GA): A line tangent to the lower border of the mandible and another line tangent to the distal border of the ramus were drawn and the angle between them was measured (Fig. 2- labeled as 1).

2. Antegonial angle (AGA): The antegonial angle was measured by the intersection of a line parallel to the anterior slope of the antegonial notch with another parallel to the posterior slope of the antegonial notch.

3. Mandibular cortical width (MCW): A line parallel to the long axis of the mandible and tangential to the inferior border of the mandible was drawn. A line perpendicular to this tangent intersecting the inferior border of the mental foramen was constructed, along which mandibular cortical width was measured (11). (Fig. 2- labeled as 3)

4. Maxillary first molar tooth length: When the palatal root was clearly visible a line between the apex of this root and groove between the mesio-buccal and disto-buccal cusps was drawn and the length of it was measured. When palatinal root was not clearly visible, then a line connecting the apices of mesio-buccal and disto-buccal roots was drawn and a line intersecting this line with right angle and beginning from the groove between the mesio-buccal and disto-buccal cusps was drawn and the length of it was measured. (Fig. 3-labelled as 1)

5. Coronal pulp width of maxillary first molar: The coronal pulp width of maxillary first molar was measured from the widest part in the horizontal plane. (Fig. 3- labeled as 2)

6. Mandibular first molar tooth length: A line was drawn passing through the apices of mesial and distal root and a second line which intersects this line with right angle beginning from the groove between mesio-buccal and disto-buccal cusps was drawn and the length of this line was measured. (Fig. 3-labelled as 3)

7. Coronal pulp width of mandibular first molar: The coronal pulp width of mandibular first molar was measured from the widest part in the horizontal plane. (Fig. 3- labeled as 4)

Density of the each panoramic radiograph as a whole and fractal dimension of the selected region of interests (Fig. 3- labeled as 5) were measured by one of the observers only once for JPEG compressed and TIFF images. Fractal dimension was calculated with Box-Counting method provided in ImageJ.

Cronbach’s alpha test was used to evaluate the intra-observer and inter-observer consistency. The mean values of the measurements were calculated separately for the two observers in repeated measurements of TIFF and JPEG compressed images. These mean values were used in the statistical analysis. Normality assumption was tested with Kolmogorov-Smirnov test. While the data of the first observer for MCW, GA, AGA in JPEG compressed images and the second observer for MCW, GA, AGA in JPEG compressed images and GA in TIFF images and density measurements in TIFF images were not having normal distribution; other measurements such as maxillary molar coronal pulp width, maxillary molar tooth length, mandibular molar coronal pulp width, mandibular molar tooth length, density of the radiographs and fractal dimension were having normal distribution. For normally distributed data paired samples t-test was used and for the data not meeting this criterion, non-parametric counterpart of this test (Wilcoxon Signed Rank test) was used. SPSS 10.0.1 for Windows was used for the statistical analysis (Rel. September 27.10.1999. Chicago: SPSS Inc.). As multiple comparisons were being made, Bonferroni adjustment (0.05/9: 0.005 in this study) was applied and for statistically significance p value was found as 0.005.

## Results

Descriptive statistics of the Cronbach’s alpha values were grouped as angular measurements (gonial and antegonial angle), vertical measurements (mandibular cortical width, maxillary and mandibular first molar tooth length), horizontal measurements (maxillary and mandibular first molar coronal pulp width) for the two observers together and density and fractal dimension measurements of one observer are given in  Table 1. It was found that the repeatability of angular measurements had the highest Cronbach’s alpha value (0.997) followed by vertical and horizontal measurements. Inter-observer and intra-observer Cronbach’s alpha values of the two observers for TIFF and JPEG compressed images and the results of the significance between groups for the two observers separately are given in  Table 2. There was statistically significant difference for both of the observers in MCW measurements between JPEG and TIFF files (1st ob. p: 0.002; 2nd ob. p: 0.003) There was statistically significant difference for the first observer AGA (1Ob p: 0.000) and maxillary molar coronal pulp width (1st ob. p: 0.000) measurements between JPEG and TIFF files. There was also statistically significant difference between the density (p: 0.000) and fractal dimension measurements (p: 0.000) of TIFF and JPEG compressed images.

**Table 1:**
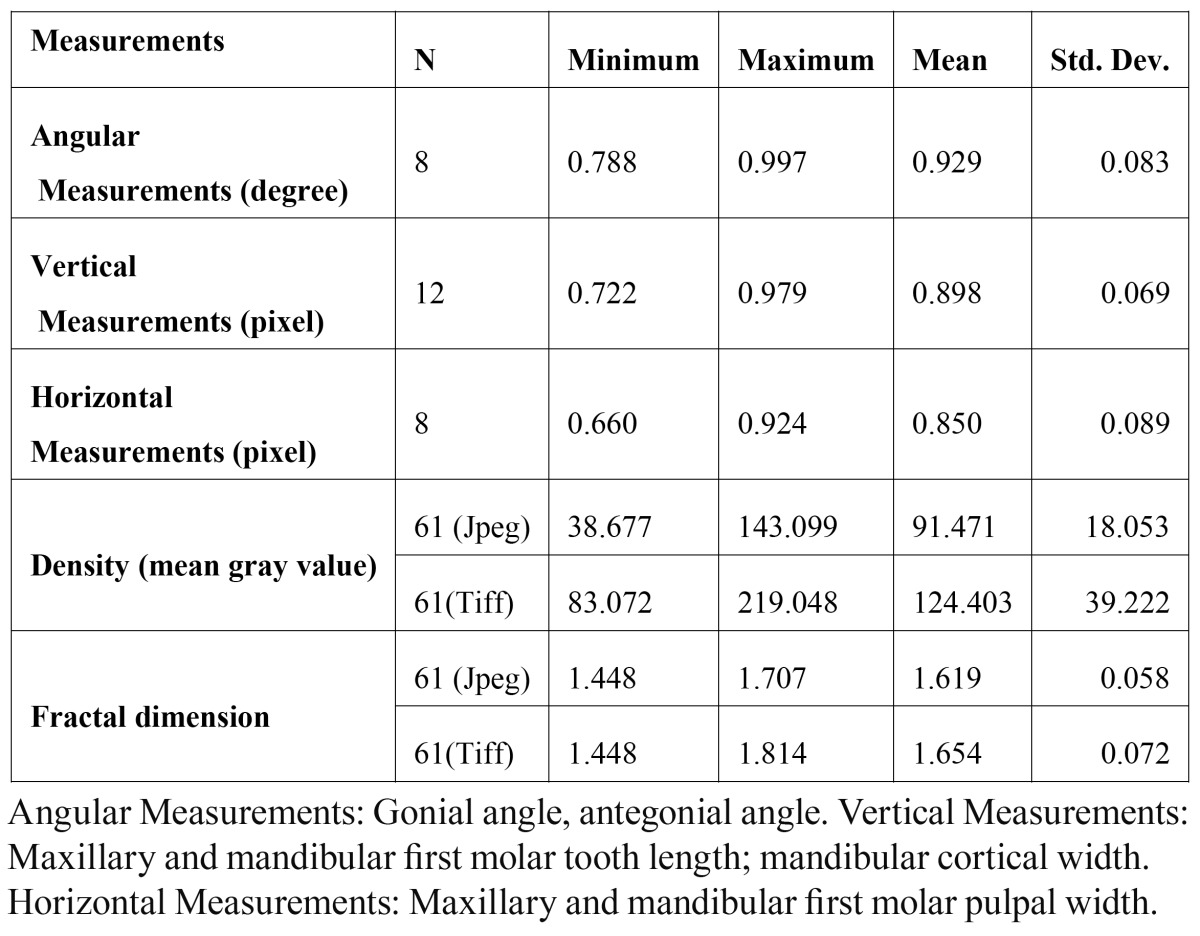
Descriptive statistics of the fractal dimension, density and Crohnbach alpha values for the two observers.

**Table 2:**
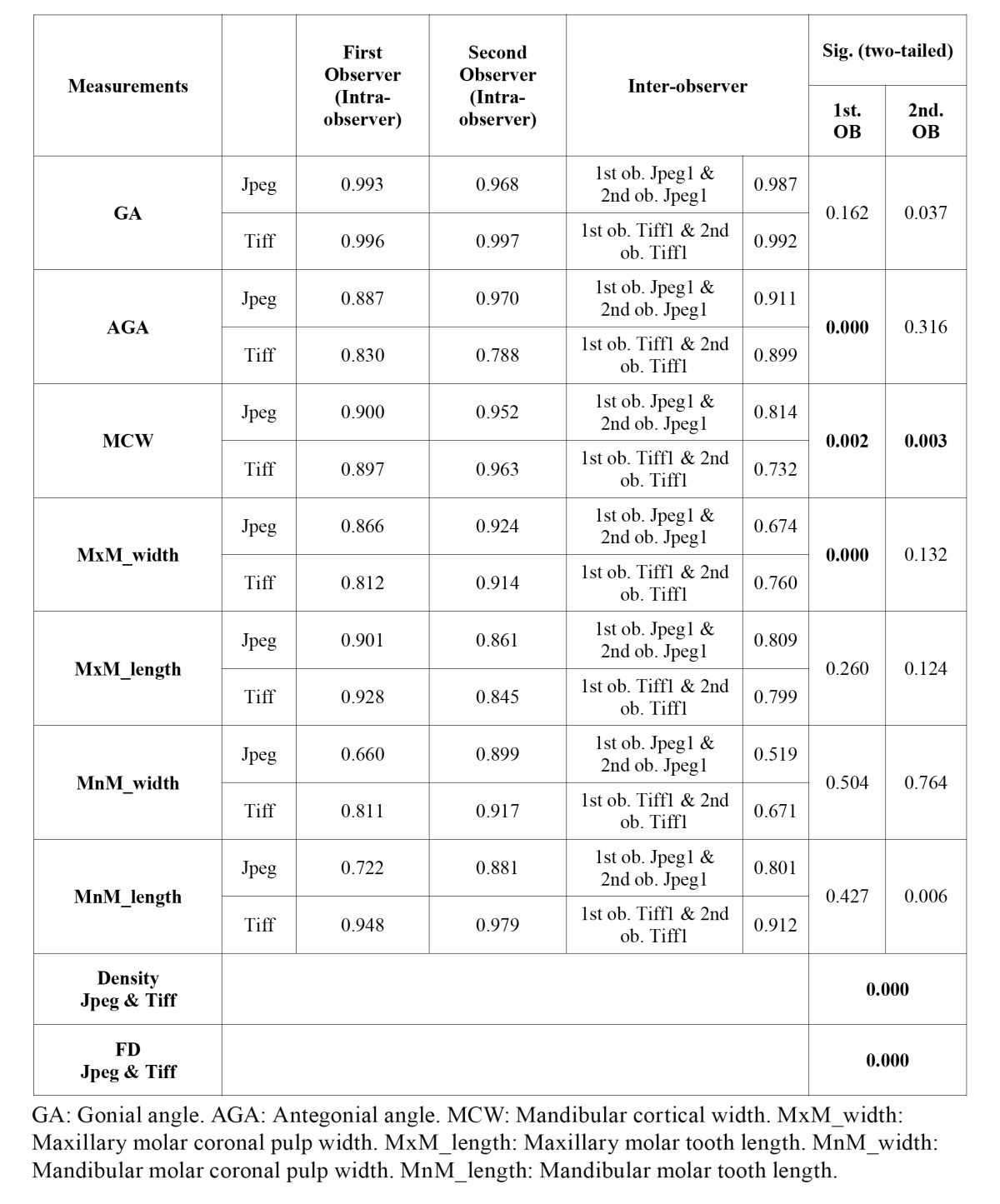
Inter-examiner and intra-examiner Crohnbach alpha values of the two examiners for Tiff and Jpeg compressed images and the results of the significance between groups for the two examiners separately.

## Discussion

In this study, different types of measurements were performed with the purpose of evaluating the effects of compression on digital panoramic radiographs. Angular measurements were performed by measuring gonial and antegonial angle; length measurements were performed in the horizontal and vertical plane. Coronal pulp widths of maxillary and mandibular first molar teeth were the measurements in horizontal plane. Length of maxillary and mandibular teeth and the mandibular cortical width are the measurements in vertical plane. Trabecular architecture has a complex structure that cannot be explained with Euclidean geometry but with fractal geometry (12). This complexity of trabecular bone was evaluated with fractal dimension in box counting method. Densities of the each panoramic radiograph as a whole in TIFF and JPEG compressed images were measured.

Radiographic image compression is performed to facilitate transmission and storage (13). Essentially, there are two types of image compression: ‘Lossless’ (reversible) and ‘lossy’ (irreversible) compression. There is no loss of information in the compressed image data in lossless compression. The use of irreversible compression is receiving more attention as a means of reducing the file size of diagnostic digital images to reduce storage and decrease image transmission times. Lossy compression offers considerably higher reduction in the file size than lossless compression, but this compression leads to a loss in image information (14). In this study the total file size of TIFF images, consisting of 61 digital panoramic radiographs was 187 MB and when these images were compressed to JPEG images via low quality with baseline optimized format, there was approximately 96% reduction in the total file size and it decreased to 7.35 MB. The effects of compression might be removal of noise at low level compressions, blurring at moderate to high levels of compression and artifacts at high levels of compression. High frequency features are usually more vulnerable to compression. Fine, irregular texture patterns such as the trabecular pattern of bone contains many small, high frequency coefficients; so it would be expected them to degrade easily (15).

The precision and accuracy of digital measurements in two modes of image display (1:1 and 2:1 magnification) in direct digital panoramic radiography was evaluated in a study (16). It was shown that digital measurements show adequate reproducibility. The most reliable measurements were obtained of linear objects in the horizontal plane. Digital measurements are reported to be sufficiently accurate for clinical use. The examiner in that study considered it to be somewhat easier to perform horizontal than vertical measurements with the mouse-driven cursor which they explained as a personal preference. In this study it was found that constructing angles on the right side of the radiographs (which is the left side of the patient) were much easier than left side of the radiographs for right handed examiners and for this reason, all measurements were performed on the right side of the radiographs. The measurements performed in the vertical plane such as the tooth length and mandibular cortical width had higher repeatability (Cronbach’s alpha: 0.898) than measurements in the horizontal plane (Cronbach’s alpha: 0.850) such as pulpal width of maxillary and mandibular molar width. Angular measurements had the highest repeatability (Cronbach’s alpha: 0.929).

The dimensions of the facial skeleton and the shape of the mandibular base, especially the gonial angle correlates with the function and shape of the muscles of mastication (17). In this study there was no statistically significant difference for gonial angle between JPEG compressed and original TIFF images for both of the observers (1st ob. p: 0.162; 2nd ob. p: 0.037). The repeatability of the gonial angle measurements for both of the observers was excellent (from 0.993 to 0.997).

The upward curving of the inferior border of the mandible anterior to the angular process is known as antegonial notching or antegonial angle. The strategic position of the shape has been considered as an indicator of how the mandibles will grow (3). Ghosh et al. found that antegonial angle decreases and the antegonial depth increases with the advancing age. Similar trends were seen when teeth are lost. Furthermore there is an inherent asymmetry in the antegonial region in right and left side (3). The repeatability of antegonial angle measurements in TIFF and JPEG compressed images for both observers were varying between 0.788 to 0.970 and although there was statistically significant difference between the measurements of antegonial angle in TIFF and JPEG compressed images for the first observer (1st ob. p: 0.000), there was no statistically significant difference for the second observer (2nd ob. p: 0.316). The repeatability of antegonial angle measurements was lower than gonial angle measurements (Cronbach’s alpha values from 0.830 to 0.970). The reason of this relatively low repeatability might be that, some of the patients’ mandibular base has a nearly flat shape and it is hard to construct the antegonial angle in these patients. This shows that although antegonial angle measurements in JPEG and TIFF image files are highly reproducible; depending on the observer, there might be statistically significant difference between measurements of JPEG and TIFF images.

In this study, the mean gray value for the TIFF and JPEG compressed images were 124.403 and 91.471 respectively. There was a decrease in density with the compression and this decrease was statistically significant (p: 0.000) between TIFF and JPEG compressed images. This means that JPEG compressed images had higher contrast than TIFF images in the studied sample of radiographs. This decrease in density may affect the observers’ decisions while making measurements. For instance they have to follow up the edges of the mandible during mandibular cortical width measurements and constructing the gonial / antegonial angles. In mandibular cortical width measurements, they also had to localize the place of mental foramen which is within the trabecular bone structure. Since the trabecular structure is thought to degrade easily as it contains many small high frequency coefficients during compression, measuring mandibular cortical width might be affected from high compression rates. There are various studies evaluating mandibular cortical width and its relation with osteoporosis. Decreasing cortical width was found to have a correlation with osteoporosis (18); however other studies found no correlation between osteoporosis and mandibular cortical width (19) .The repeatability of mandibular cortical width measurements were good to excellent and there were statistically significant difference between the mandibular cortical width measurements in TIFF and JPEG compressed images for both of the observers (1st ob. p: 0.002 and 2nd ob. p: 0.003).

Maxillary first molar coronal pulp width, mandibular first molar coronal pulp width, maxillary first molar tooth length and man-dibular first molar tooth length are measured in the horizontal (or a little bit oblique) and vertical (or a little bit oblique) plane. These kinds of measurements are used in dental age calculation with radiographs (5). In this study, the repeatability of the length measurements in vertical plane were varying between 0.845 to 0.928 for maxillary first molar tooth length and 0.722 to 0.979 for mandibulary first molar tooth length. There was no statistically significant difference for the maxillary and mandibular molar tooth length in TIFF and JPEG compressed images for both of the observers however there was statistically significant difference between the measurements of maxillary first molar coronal pulp width between TIFF and JPEG compressed images (1st ob. p: 0.000) for the first observer. The repeatability of the measurements of maxillary molar coronal pulp width of the first observer was also lower than the second observer ( Table 2). The reason of this might be the poor localization of the widest place of the pulp chamber by the first observer.

Fractal dimension (FD) can be viewed as a measure of irregularity of many physical processes (12) and it finds a lot of application areas in dental research. Trabecular bone structure was evaluated with fractal dimension in various diseased conditions (20,21). Each structure feature will show a correlation with density and complex objects have a higher fractal dimension (12). In this study the mean fractal dimension in TIFF images (mean FD: 1.654) was higher than in JPEG compressed images (mean FD: 1.619). Our results are consistent with loss of image detail during compression. Fractal dimension showed a statistically significant decrease in JPEG compressed images (p< 0.001).

The experience of the observers was widely different from each other. The first observer was working in maxillofacial radiology department for more than 10 years and the second observer was working in the maxillofacial radiology clinic for one year. However, the second observer was very successful in the repeated measurements. The first observer’s Cronbach’s alpha values were in a range of 0.996 and 0.660. The second observer’s Cronbach’s alpha values were in a range of 0.997 and 0.788. In a study, general dental practitioners and fourth year dental students diagnostic accuracy for proximal dentine caries using different measures was compared with simulated bitewing radiographs (22). The students were found to have better sensitivity but worse specificity than dentists. In that study, the simulated bitewing radiographs composed of extracted and cleaned molar and premolar teeth. After the radiographs had been taken, the teeth were sectioned through the deepest point of any lesion present and microradiographed. In this study reliability of the measurements were evaluated however it was not possible to evaluate the specificity. In order to evaluate specificity, dry skulls should be used and sectioned, however panoramic radiographs of patients were used in this study.

As a conclusion; when the Cronbach’s alpha values of measurements were grouped according to their orientation, it was found that angular measurements had the highest repeatability (Cronbach’s alpha: 0.929) followed by vertical (Cronbach’s alpha: 0.898) and horizontal measurements (Cronbach’s alpha: 0.850). Mandibular cortical width is found to be affected from compression as there is statistically significant difference between TIFF and JPEG compressed images for both of the observers. For the other measurements such as antegonial angle and maxillary molar coronal pulp width, in which there was statistically significant difference between the TIFF and JPEG compressed images for the first observer, observer dependent factors might be effective. During JPEG compression; some information within the image is lost and as a consequence of this, it is not surprising to find a statistically significant difference between TIFF and JPEG compressed images for fractal dimension and density.

## References

[1] Stramotas S, Geenty JP, Petocz P, Darendeliler MA (2002). Accuracy of linear and angular measurements on panoramic radiographs taken at various positions in vitro. Eur J Orthod.

[2] Shahabi M, Ramazanzadeh BA, Mokhber N (2009). Comparison between the external gonial angle in panoramic radiographs and lateral cephalograms of adult patients with Class I malocclusion. J Oral Sci.

[3] Ghosh S, Vengal M, Pai KM, Abhishek K (2010). Remodeling of the antegonial angle region in the human mandible: a panoramic radiographic cross-sectional study. Med Oral Patol Oral Cir Bucal.

[4] Devlin H, Allen P, Graham J, Jacobs R, Nicopoulou-Karayianni K, Lindh C et al (2008). The role of the dental surgeon in detecting osteoporosis: the OSTEODENT study. Br Dent J.

[5] Kvaal SI, Kollveit KM, Thomsen IO, Solheim T (1995). Age estimation of adults from dental radiographs. Forensic Sci Int.

[6] Yasar F, Yesilova E, Akgünlü F (2010). Alveolar bone changes under overhanging restorations. Clin Oral Investig.

[7] Yasar F, Akgünlü F (2005). Fractal dimension and lacunarity analysis of dental radiographs. Dentomaxillofac Radiol.

[8] Fidler A, Likar B and Skaleric U (2006). Lossy. JPEG compression: easy to compress, hard to compare. Dentomaxillofac Radiol.

[9] Angelopoulos C, Bedard A, Katz JO, Karamanis S, Parissis N (2004). Digital Panoramic Radiography: An Overview. Semin Orthod.

[10] Fidler A, Skaleric U, Likar B (2007). The effect of image content on detail preservation and file size reduction in lossy compression. Dentomaxillofac Radiol.

[11] Taguchi A, Sugino N, Miki M, Kozai Y, Mochizuki N, Osanai H et al (2011). Detecting young Japanese adults with undetected low skeletal bone density using panoramic radiographs. Dentomaxillofac Radiol.

[12] Geraets WG, van der Stelt PF (2000). Fractal properties of bone. Dentomaxillofac Radiol.

[13] Krupinski EA, Williams MB, Andriole K, Strauss KJ, Applegate K, Wyatt M et al (2007). Digital radiography image quality: image processing and display. J Am Coll Radiol.

[14] Seeram E (2006). Irreversible compression in digital radiology. A literature review. Radiography.

[15] Rabbani M, Jones PW (1991). Image Compression Techniques for Medical Diagnostic Imaging Systems. J Digit Imaging.

[16] Schulze R, Krummenauer F, Schalldach F, d'Hoedt B (2000). Precision and accuracy of measurements in digital panoramic radiography. Dentomaxillofac Radiol.

[17] Xie QF, Ainamo A (2004). Correlation of gonial angle size with cortical thickness, height of the mandibular residual body, and duration of edentulism. J Prosthet Dent.

[18] Devlin H, Horner K (2002). Mandibular radiomorphometric indices in the diagnosis of reduced skeletal bone mineral density. Osteoporos Int.

[19] Drozdzowska B, Pluskiewicz W, Tarnawska B (2002). Panoramic- based mandibular indices in relation to mandibular bone mineral density and skeletal status assessed by dual energy X-ray absorptiometry and quantitative ultrasound. Dentomaxillofac Radiol.

[20] Demirbaş AK, Ergün S, Güneri P, Aktener BO, Boyacioglu H (2008). Mandibular bone changes in sickle cell anemia: fractal analysis. Oral Surg Oral Med Oral Pathol Oral Radiol Endod.

[21] Ergün S, Saraçoglu A, Güneri P, Ozpinar B (2009). Application of fractal analysis in hyperparathyroidism. Dentomaxillofac Radiol.

[22] Mileman PA, van den Hout WB (2002). Comparing the accuracy of Dutch dentists and dental students in the radiographic diagnosis of dentinal caries. Dentomaxillofac Radiol.

